# Addressing the needs of traumatic brain injury with clinical proteomics

**DOI:** 10.1186/1559-0275-11-11

**Published:** 2014-03-28

**Authors:** Sean Shen, Rachel R Ogorzalek Loo, Ina-Beate Wanner, Joseph A Loo

**Affiliations:** 1 Department of Chemistry and Biochemistry, University of California-Los Angeles, Los Angeles, CA 90095, USA; 2 Department of Biological Chemistry, David Geffen School of Medicine at UCLA, University of California-Los Angeles, Los Angeles, CA 90095, USA; 3 Molecular Biology Institute, University of California-Los Angeles, Los Angeles, CA 90095, USA; 4 Semel Institute for Neuroscience and Human Behavior, David Geffen School of Medicine at UCLA, University of California-Los Angeles, Los Angeles, CA 90095, USA

**Keywords:** Traumatic brain injury, Biomarker, Clinical proteomics, Mass spectrometry, Multiple reaction monitoring

## Abstract

**Background:**

Neurotrauma or injuries to the central nervous system (CNS) are a serious public health problem worldwide. Approximately 75% of all traumatic brain injuries (TBIs) are concussions or other mild TBI (mTBI) forms. Evaluation of concussion injury today is limited to an assessment of behavioral symptoms, often with delay and subject to motivation. Hence, there is an urgent need for an accurate chemical measure in biofluids to serve as a diagnostic tool for invisible brain wounds, to monitor severe patient trajectories, and to predict survival chances. Although a number of neurotrauma marker candidates have been reported, the broad spectrum of TBI limits the significance of small cohort studies. Specificity and sensitivity issues compound the development of a conclusive diagnostic assay, especially for concussion patients. Thus, the neurotrauma field currently has no diagnostic biofluid test in clinical use.

**Content:**

We discuss the challenges of discovering new and validating identified neurotrauma marker candidates using proteomics-based strategies, including targeting, selection strategies and the application of mass spectrometry (MS) technologies and their potential impact to the neurotrauma field.

**Summary:**

Many studies use TBI marker candidates based on literature reports, yet progress in genomics and proteomics have started to provide neurotrauma protein profiles. Choosing meaningful marker candidates from such ‘long lists’ is still pending, as only few can be taken through the process of preclinical verification and large scale translational validation. Quantitative mass spectrometry targeting specific molecules rather than random sampling of the whole proteome, e.g., multiple reaction monitoring (MRM), offers an efficient and effective means to multiplex the measurement of several candidates in patient samples, thereby omitting the need for antibodies prior to clinical assay design. Sample preparation challenges specific to TBI are addressed. A tailored selection strategy combined with a multiplex screening approach is helping to arrive at diagnostically suitable candidates for clinical assay development. A surrogate marker test will be instrumental for critical decisions of TBI patient care and protection of concussion victims from repeated exposures that could result in lasting neurological deficits.

## Introduction

A general goal of "proteomics" is to comprehend the relationship between the body’s proteins and how they change by disease to understand human pathophysiology, and ultimately to provide therapeutic and diagnostic tools. The completion of the human genome provided researchers with the blueprint for life; proteomics offers the potential means for analyzing the expressed genome. Proteomics attempts to determine how genes function within the genome and how they communicate with each other to (hopefully) lead to important new insights into disease mechanisms. The potential of proteomics to advance biomedical research is high because the key functional components of biochemical systems and the cellular targets of therapeutic agents, namely proteins, are being studied. Mapping proteomes from injured tissues, cells and biofluids can potentially reveal new protein targets to explore mechanisms of insults and to provide candidate lists for new disease indicators or injury biomarkers as diagnostic or prognostic tools for the clinic.

A biomarker could be simply a molecule, such as a protein whose presence or abundance in a biological sample signals a disease or insult to an organ. Thus, they are quantifiable molecules that indicate a pathophysiological process. A biomarker in accessible body fluids or tissues could greatly enhance our ability to identify patients at risk, with invisible wounds or predict outcome of serious injury. A sensitive and specific disease or injury marker such as an early protein abnormality could provide a warning sign prior to being symptomatic, and hence could result in more effective preventative care or treatment options to improve outcome.

### The challenges of clinical proteomics and biomarkers

The goal of clinical proteomics to discover new disease or injury biomarkers is challenging. Beyond the number of human genes coding for proteins, proteins are processed and modified, comprising an important dimension of information to which present proteomic technologies have but limited access. The total mRNA population, accounting for alternate splicing, RNA editing, and use of alternate promoters could contain 250,000 transcripts, while various protein modifications could increase the size of the human proteome to over 500,000 members [1]. Cellular proteins and their post-translational modifications (PTMs) change with the cell cycle, environmental conditions, developmental stage, and metabolic state. Independent of these variables, biomarkers should reliably detect changes in health status, a specific disease, or indicate whether an insult like a toxic exposure or trauma has occurred. Clearly, we need proteomic approaches that advance beyond identifying proteins to elucidating their co- and post-translational modifications, to following the dynamics of those modifications, and to linking those modifications to specific diseases or cellular responses to an insult that inflicted an organ.

Despite all of the significant advances in technologies in proteomics since its inception in the mid-1990s, with the development of more sensitive mass spectrometry detectors and more selective and specific strategies for sample processing and handling, no clinically validated disease biomarker has been discovered by proteomics to date [2].

### Meeting the challenge with targeted screens, focused selection strategies, and clinical validation

What are the major factors that hindered finding robust disease and injury biomarkers and how can these be overcome? The complexity of clinical samples themselves is a significant limiting factor. Plasma and serum, i.e., blood, have been biofluids of choice for measuring levels of proteins and other biomolecules for clinical testing, as they can be sampled noninvasively. Plasma is a protein-rich information source containing what blood circulation has encountered on its journey throughout the body and tissue perfusion. The tremendous *analytical challenge* of the large number of plasma proteins lays in their unbalanced abundance: albumin constitutes over 50% of the plasma proteins (at 30–50 mg/mL) and the most abundant 22 proteins in plasma represent approximately 99% of the total protein content in plasma leaving the majority of proteins at very low abundance. The estimated dynamic range of protein concentrations in human plasma may be up to 12 orders of magnitude [3].

**Figure 1:**
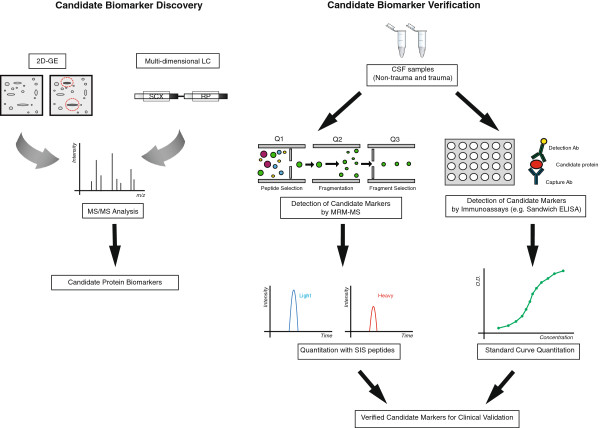
**Candidate biomarker discovery and verification workflow.** Bottom-up proteomics strategies, such as shotgun proteomics (multidimensional LC-MS/MS) and 2D-PAGE/MS, can be applied to identify putative candidate markers (left). Candidate protein markers can be subsequently verified and confirmed by targeted proteomics using standard ELISA methods or multiple reaction monitoring (MRM)-MS (right). MRM-MS offers the advantages of an antibody-independent platform with capabilities for multiplexing.

Disease or insults trigger acute events, secondary and chronic sequelae, including inflammation, wound healing, and adaptive changes that the compromised body undergoes in response to the unhealthy state. In an effort to identify original disease causes or injury factors a simple experimental model can facilitate a targeted screen circumventing secondary, less disease-specific events. As such, scientific experimental model design follows controlled strategies for reproducibility and simplicity that can facilitate the initial discovery by limiting candidate markers to those proteins that are related to a disease origin or injury cause [4,5]. One common proteomics workflow involves a 2-dimensional separation prior to protein identification to reduce sample complexity (Figure 1). Proteins can be sorted by charge (isoelectric point) and size using two-dimensional polyacrylamide gel electrophoresis (2D-PAGE) and can be enzymatically digested within the gel matrix. Despite being developed over 3 decades ago [6,7], 2D-PAGE remains one of the most powerful separation techniques for proteomic workflows and was instrumental in early protein biomarker research. Following separation, gels are stained and differentially expressed protein spots excised, enzymatically digested with trypsin, and identified by MS requiring only sufficiently accurate mass measurements (low part-per million range) performed on one or two tryptic peptides to identify silver-stained protein spots [8].

A second strategy advocates first enzymatically (e.g., with trypsin) or chemically cleaving ("breaking") a complex mixture of cellular proteins, and then "sorting" the peptides by one or more steps of chromatography. MS analyzes the recovered fragments as in the previous approach, and software matches the fragments to the proteins from which they are derived. Examples of this experimental approach include multidimensional protein identification technology (MudPIT) that couples two or more dimensions of chromatographic separations, e.g., strong cation exchange (SCX) with reversed-phase chromatography [9,10]. While the outlined approaches have been instrumental in biomarker discovery research, the extensive sample preparation and time required in gel fractionation and long HPLC LC-MS/MS analyses make discovery proteomics feasible for only limited numbers of samples per project [11,12]. A simplified disease or injury model using a controlled experimental design may help to relieve a proteomic screen from confounding complexities of clinical samples [4,13-15].

A straightforward *selection* of suitable marker candidates from the ‘long list’ of identified injury or disease specifically changed proteins should arrive at a manageable ‘short list’ of possible disease marker candidates. A tailored selection strategy will consider injury cause, marker candidates with the necessary reporting power for the cause as well as organ specificity and exclusion of proteins normally present in healthy plasma and tissues.

The subsequent validation of selected disease or injury markers from a group of candidates may occur stepwise starting with a preclinical smaller cohort of patients and controls, allowing to test for normality [16]. Following initial confirmation, a larger subject cohort can be enrolled in clinical trials allowing for receiver operating characteristic curve analyses that will establish the basis for biomarker suitability in the clinic [17]. Currently, the majority of biomarker validation studies have been performed by enzyme-linked immunosorbent assay (ELISA). This highly sensitive method is limited for use early in the verification process, as antibody pairs have to be optimized for specificity and sensitivity for each marker separately. As mass spectrometry measurements improve in sensitivity to match immunoassay detection limits (pg/mL), a targeted and quantitative mass spectrometry application can provide multiplex capacity and absolute specificity by gas-phase sequence determination, making it an ideal alternative for assessing validity of selected marker candidates.

### The need for markers of Traumatic Brain Injury (TBI)

Neurotrauma to the central nervous system (CNS) is a serious public health problem in the US; among US civilians, TBI is most common in infants and toddlers, adolescents and the elderly [18]. The US National Institute of Neurological Disorders and Stroke estimates that 2.5-6.5 million Americans have had one or multiple TBIs [19]. In the US military there were over 212,000 service men and women diagnosed with some form of TBI between January 2000-May 2011, roughly accounting for one-third of all injured US soldiers, making TBI the *signature injury* of the wars in Iraq and Afghanistan compared to past wars [20]. TBI contributes to over one-third of all injury-related deaths, yet 75-90% of all brain trauma cases are considered to be *mild TBI* (mTBI), many without visible wounds *that often are undiagnosed*[21]. *Better diagnostic tools are needed to detect head injuries, especially mTBI, to confirm and to monitor the severity of TBI in order to determine the best course of action acutely and later post-injury.* The neurotrauma field has currently still no chemical diagnostic marker in clinical use. Here we will outline briefly the spectrum of TBI and give examples where a surrogate chemical marker assay for TBI would be of great benefit to patients, high risk populations, their families and doctors.

Head injuries can be classified into penetrating and non-penetrating TBI. Penetrating TBI involves physical compromise of the skull by an external object resulting in specific, focused injury most commonly characterized by hemorrhages and lesions. Non-penetrating TBI, is much more difficult to assess, as injuries may not be visible or located precisely. Closed head injuries are caused by rapid acceleration and deceleration of the brain within the skull and inflict shear and deformation forces on gray matter tissue and white matter tracts [22]. Each trauma patient is a unique injury case with individual complexity, thus the field distinguishes mainly between severe and mild TBI (mTBI) as opposite ends of a clinical spectrum of manifestations. Evaluating and predicting outcome in severe TBI is often problematic, especially for patients without visible wounds such as infants.

Diagnostic neurotrauma tools include imaging techniques, neurocognitive examinations, and for severe TBI patients, the determination of post-traumatic amnesia, but they provide only estimates of the dynamically evolving injury process. Functional MRI (fMRI) and the detection of regional blood flow changes (e.g., PET scans) are not always available, cannot be obtained in critically ill patients, and are not definitive. Radiological brain scans on infants and toddlers are widely considered problematic because the radiation dose endangers the developing brain. Absence of imaging in the pediatric clinical praxis prevents distinguishing brain injury from frequent intestinal flu or even infant irritability [23]. Non-accidental head injury, or "Shaken Baby" syndrome, caused by rotation-acceleration strains on the brain in the still loosely connected infant skull causes bleeding and swelling that can lead to catastrophic intracranial damage and can severely impair normal brain development (and can even lead to death) [23]. Undiagnosed victims may be sent back to continued abuse. On the other hand, imaging does not distinguish inflicted head injury from non-traumatic bleeding, originating from a trauma independent condition – a situation in which legal authorities, parents and care-givers would greatly benefit from an assay for brain trauma-specific chemicals [24,25]. Mechanical impacts traumatizing the brain obviously need to be clinically differentiated from trauma in other organs or from other, non-traumatic brain injuries like stroke, ischemia, bleeding diseases, poisoning, epilepsy or chronic degenerative diseases for proper treatment and activities in the operating room and the courtrooms [26]. Monitoring daily progression of a severe TBI patient by repeated imaging can be quite impractical, considering life supporting intensive care instrumentation. A fluid derived chemical marker for compromised brain cell viability will be a useful added measure of the patients evolving status and could aid in outcome prognosis.

For the vast majority of mTBI/concussion patients, there are no objective diagnostic or prognostic tools [27]. A ready diagnostic tool at point of care acutely after TBI is needed especially for high-risk individuals (e.g., athletes, military personnel). An objective and unambiguous trauma biochemical assay would be valuable for legal authorities in forensic cases that currently rely on neuropsychological testing that lacks premorbid base rates and is subject to malingering and subjective interpretation. Thus, for high-risk groups, for mild and severe TBI cases as well as for all pediatric neurotrauma patients, there is an urgent need for an accurate, unambiguous chemical measure indicating that a significant impact to the brain had occurred.

Moreover, a *second* hit to a concussed, vulnerable brain can, in rare cases, have a catastrophic outcome with permanent brain damage or even death (known as the *second impact syndrome*) [28]. Several repeated concussions over time can in later years cumulate in irreversible brain damage with devastating psychological and cognitive decline, a pathological condition now defined as chronic traumatic encephalopathy (CTE) [29,30]. Military personnel and veterans with mild TBI often suffer from post-traumatic stress disorder (PTSD) after being exposed to blast waves from explosive devices [31,32].

Certain areas of the brain may be more susceptible to concussive trauma. A recent study investigated longitudinal changes in global and regional brain volume in patients one year after mTBI and correlated such changes with clinical and neurocognitive metrics. Magnetic resonance imaging data showed measureable global brain atrophy, larger than that in control subjects one year after mTBI. Atrophy was found in specific regions of the cingulate cortex independent of the site of initial trauma. The cingulate cortex’s role in rational cognitive functions such as empathy, impulse control, and emotion correlate strongly with the patient’s observed clinical symptoms of increased depression and anxiety [33]. These finding are supported by an independent study of National Football League players and referees using positron emission tomography (PET) with a tau specific tracer that showed higher densities of tau tangles in regions of the brain involved in a nearby region (caudate nucleus) that is also associated with learning, memory, emotion, and language comprehension. The deposition of tau tangles is consistent with those observed in CTE autopsy patients [34].

Current evaluation of concussion is basically an assessment of neurocognitive deficits, often not immediate and requires extensive neuropsychological testing that is subject to motivational confounds, while critical care treatment decisions have to be made immediately by emergency clinical personnel and surgeons. Severity classification of TBI patients relies on assessing the level of consciousness, commonly with the Glasgow Coma Scale (GCS), which is an insensitive measure. Testing relies on verbal communication, and proper motor control and eye function, which are often impaired after TBI. Brain function-altering substances such as drugs, alcohol, pain medication, sedatives, or even induced coma as part of emergency and intensive care routine obviously compromise the use of memory recall and the GCS. Although predictors of TBI exist, such as the Standardized Assessment of Concussion test, these tools offer little insight into the pathology of the disease beyond determining whether a concussion has occurred or not. Because of this lack of insight into TBI, licensed health care providers of concussive sports injury are conservative in their approach to player safety after injury with the hope that coordination between sideline and clinical practitioners will aid in improving our understanding of the extent of impairment for various types of sports related concussions [35].

### Current potential TBI biomarker candidates

An ideal biomarker should be both specific to head trauma as well as sufficiently sensitive to be measured and quantified reproducibly in patient blood or other peripheral or proximal fluid samples (such as CSF) by an assay of choice. These markers should be acutely released into the fluids following injury and show a distinct temporal signal pattern. The identification of a unique TBI biomarker(s) or surrogate brain cell injury markers that meet these criteria would provide physicians with an objective method for early diagnosis of brain injury and enable early assessment of severity, intervention, and monitoring disease progression [36]. Multiple neurotrauma signature markers would allow for correlation analyses with improved statistical power using multivariant logistic regression or similar analyses [37]. Finding candidate TBI markers is pursued typically by these strategies: (1) Classical deduction chooses proteins with literature reported association to brain injury or its secondary events like inflammation, axonal degeneration or reactive astrogliosis. (2) Hypothesis driven animal trauma model studies report changes in specific proteins using available antibodies or pathway tailored kits [38]. (3) Discovery of trauma associated proteins using a proteomic screen of samples derived from animal injury models or small patient cohorts [39-43]. Surprisingly, few screens address the impact of mechanical trauma on brain cells, i.e., cell death [13,14,44]. After briefly summarizing currently investigated candidate TBI markers, we will evaluate challenges and alternatives in identifying TBI markers.

#### Inflammatory markers

Part of the pathology of CNS injury is characterized by secondary effects, including the inflammatory response to TBI. Cytokines are key mediators in the process of (neuro)inflammation [45] and increased concentrations of these compounds have been associated with severe CNS injury as well as post-traumatic hypoxia [46,47]. For example, elevated levels of interleukin-10 (IL-10), an anti-inflammatory cytokine, was measured in low pg/mL levels in CSF and low-mid pg/mL levels in serum (Table 1) and correlated with severe TBI determined by the GCS [46,48-50]. Higher Il-10 serum levels illustrate the systemic nature of an inflammatory response. Such responses are systemic in nature and not specific to TBI, but occur with any insult, hence inflammatory markers are not ‘pointing to brain injury’.

**Table 1:** **Candidate marker biofluid concentrations**^**a**^

**Surrogate marker**	**Process or source, cell type**	**Concentration in TBI biofluids (ng/mL)**	
		**CSF**	**Serum**
IL-10	Inflammation	0.001-0.060 (children, [48])	0.050-0150 [49]
		0.002-0.005 (adult, [46])	
S100B	Astroglia	1.0-15.0 [51]	0.01-0.70 [46,52,53]
GFAP	Astroglia	9.0-22.0 [54]	0.14-15.0 [55]
NFL/NFH/P-NFH	Axon	0.13-2.5 and 49–562 [56,57]	NA
MBP	Axon/oligodendrocytes	NA	0.50-100.0 [46,58]
Tau/amyloid β	Axon	Tau: 0.035-5.72 (neonate, [59])	0.91- 5.1 [60,61]
		1,519.6 – 2,308 (adult, [62-66])	
		Aβ 1–42: 1.17 (adult, [67]	
NSE	Neuron	10-30 [46]	10-20 [58]
UCH-L1	Neuron	20-300 [68]	1.0-15 [68]
α-spectrin-II BDP	Neuron + astroglia	0.0-100 [69]	NA
MAP-2	Neuron	NA	0.04-0.06 [70]

^a^Selected examples of reported TBI markers for which some concentrations were found; NA – not available.

#### Neuronal markers

With their elongated axonal and dendritic processes, neurons are exposed to shear forces associated with the whiplash trauma of a concussion. Acute plasma membrane permeablility, or mechanoporation, compromises cell integrity and is linked to diffuse axonal injury in response to a mechanical impact [71-73].

Tau protein is a member of microtubule-associated proteins involved in maintaining cytoskeletal structure and axonal transport. It is expressed by CNS neurons and oligodendrocytes and found primarily in axons [74]. Traditionally used in the diagnosis of Alzheimer’s disease, elevated levels of Tau in CSF and serum have been linked to CNS insults like TBI and stroke [62,75]. CSF and serum studies of TBI patients have measured elevated Tau protein concentrations in the 1000 ng/mL range in young adult TBI patients, whereas it is three orders of magnitude lower in neonates with brain insults [59,65,66]. Because of Tau’s chronic accumulation after various CNS insults, it seems less useful as an acute head trauma marker.

Mylein basic protein (MBP) is released with myelin debris that accumulates with axonal damage in the injured brain or spinal cord. MBP is one of three proteins comprising the myelin sheath essential for axonal impulse conduction [76]. MBP markers have shown promise in the appraisal of TBI with serum levels in the low-mid ng/mL range [77,78]. Similar to GFAP (*vide infra*), studies have demonstrated degradation of MBP isoforms as a result of TBI [79,80].

Neuron Specific Enolase (NSE), Microtubule-associated protein 2 (MAP-2) and ubiquitin C-term hydrolase L1 (UCH-L1) all display differential expression patterns in TBI patients. NSE, a glycolytic enzyme isoform of neurons, has been documented to increase following head trauma [77], but has a slow elimination process, making it difficult to distinguish between primary and secondary injuries [81]. Additionally, NSE is released during the process of hemolysis, making it difficult to pin down the source of injury [82].

Microtubule-associated protein 2 (MAP-2) is a cytoskeletal-associated protein localized to dendrites of neurons that is believed to function in the growth and maturation of dendrites as well as cytoskeletal organization [83]. Previous studies have demonstrated that MAP-2 is absent from damaged regions of the brain and that serum levels increase early after injury [84]. Mondello *et al.* assessed the long-term release of MAP-2 in blood 6 months post trauma by ELISA immunoassay and found that severe TBI patients had significantly higher serums levels of MAP-2 compared to normal non-TBI patients. TBI patients in a vegetative state, as assessed by the GCS, however, showed no increase in serum MAP-2 versus controls. This suggests that MAP-2 could provide insight into the mechanism of neuronal remodeling as well as discriminate between patients with deficits in consciousness and increased risk of unfavorable outcomes [70].

Ubiquitin C-terminal hydrolase-L1 (UCH-L1) has been identified in a cell death culture assay and is verified by ELISA to be significantly increased in TBI patients [85]. Neurodegenerative marker UCH-L1 fluid levels are also elevated in ischemia, vasospasm, infarction, and carbon monoxide poisoning [86-88]. UCH-L1 is a proteolytically stable, abundant neuronal protein [69,70,85,89]. Future studies will show whether these proteins would be present in mTBI subjects without significant brain cell death.

#### Trauma specific breakdown products of neuronal and glial cytoskeletal proteins

Spectrin breakdown products (SBDPs) have been identified as potential TBI biomarkers in rat CSF fluid [90]. αII-Spectrin is the submembraneous cortical cytoskeleton of neurons and astroglia, sharing 50-59% homology with the abundant erythroid α-spectrin [44,91]. Cell-death associated spectrin fragments of molecular weight 150 kDa (SBDP150) and two N-terminal fragments at 145 kDa (SBDP145) and 120 kDa (SBDP120) cleaved by calpain and caspase-3 have been identified in a cell death culture model [87,92,93]. Using a sandwich ELISA methodology, Mondello *et al.* showed both SBDP145 and SBDP120 increased in patients post-TBI, with SBDP145 present immediately post-trauma and SBDP120 most accurately measured 24 hr post-injury. SBDP CSF levels greater than specific thresholds were shown to correlate with poor outcome and mortality and the temporal expression of SBDP for non-surviving patients differed from that of surviving patients. Thus, if cross-reactivity and breakdown specificity is controlled, SBDPs in CSF may aid to predict the severity of injury and mortality [69].

#### Astroglial markers

Astroglia are the most abundant cells in the human cerebral cortex [94] and respond to insult by becoming reactive, a process that involves gene expression, morphological changes, proliferation, and the formation of a glial scar around lesions [95-99]. However, astrocytes are also trauma victims as they are especially vulnerable to acidosis, pressure elevation, and hypoxic/ischemic damage, known co-morbidities of TBI [100-103]. Human astrocytes display very long thin processes that cross through several laminae from the pia to the ventricular walls, so called interlaminar processes and are hence vulnerable to shear and deformation forces similar to those that cause diffuse axonal injury in white matter tracks [104,105]. Two of the most well studied TBI marker candidates are S100β and glial fibrillary acidic protein (GFAP), both glial proteins. S100β is a calcium binding protein that is predominantly produced by astrocytes within the CNS. Because S100β is also produced in a variety of non-CNS cells (e.g., lymphocytes, bone marrow, adipocytes , and glia of peripheral nerves), brain specificity is its problem [106]. It has, however, been reported that the few extracranial sources of S100β are short lived compared to S100β from cerebral lesions [107,108]. Elevated S100β concentrations have been measured in the ng/mL and pg/mL range in TBI patient CSF and serum, respectively [51,53]. Despite the immediate spike in S100β levels, it has been found that S100β measurements taken 24 hours post TBI offer the most prognostic value for patient outcome due to initial interference from external S100β [52]. S100β is released into the perivascular space immediately following blood brain barrier (BBB) compromise and may serve as a BBB-permeability marker [109]. Additionally, higher levels of S100β have been correlated to patients suffering from post traumatic hypoxia, demonstrating the interrelation between secondary effects and amplified biomarker expulsion [46].

GFAP is an intermediate filament that is highly enriched in CNS astroglia, but is also expressed in Schwann cells and olfactory ensheathing glia of peripheral nerves [110-112]. GFAP levels are persistently elevated after *severe* TBI in CSF and serum, relate to poor outcome, and are predictive for mortality [54,55,113]. Serum levels of GFAP show high variability or no elevation after mTBI, yet reports are confounded by varying delimitation of ‘mild’ as to include more moderate cases with lesions and positive imaging signals or not. Thus the discriminative power of GFAP as a mild neurotrauma biomarker is conflicted [56,114]. Measured CSF levels of several biomarkers in boxers acutely after one or repeated blows to the head as well as after 14 days, revealed elevated levels of GFAP with large variations among the boxers suffering a concussion [56]. GFAP breakdown products are found after TBI and are being explored as insult-specific markers [114-116].

### Strategies for addressing the challenges in identifying and validating new TBI biomarkers

For a brain cell specific protein to be a trauma marker, either it should be selectively expressed in response to the trauma and then discharged into fluids, or cytosolic proteins released solely from injured neurons and glia with compromised membrane integrity or dying brain cells [73,117]. A suitable study design to identify fluid-derived trauma specific proteins would employ a targeted proteomic screen on a defined trauma model. Experimental animal injury models were developed with the effort to mimic human TBI as closely as possible while underlying cellular and molecular mechanisms of acute trauma are still scantly investigated. The predominant criterion is to recapitulate the clinical manifestation of TBI over studies using simplified reproducible trauma models with the goal to determine primary cellular injury consequences [4,5]. Most commonly used injury models include focal injuries with the animal’s head in a fixed position, like fluid percussion and controlled cortical impact, which produce a focal contusion with hematoma and hemorrhage while the dura remains intact [118,119]. Also used is Marmarou’s weight drop model where distributed forces cause diffuse injury with the animal’s head unrestrained in a helmet and the brain is therefore subjected to rotational forces as well [120,121]. Blast injury models historically use shock tubes and larger animals, but have been adapted recently to rodents as well as investigated for milder blast effects from explosion exposures in the field [122].

### Developing biochemical markers of TBI by proteomics and mass spectrometry

Proteomic studies of injured brain or spinal cord tissue are being done in these injury models and are providing lists of protein changes that are difficult to interpret due to the complexity of events at and around a dynamically changing lesion site and variations between models [39,40,42]. Injury zones are not reproducibly defined from lab to lab as histopathological analyses have for long not followed standardized analysis and reporting criteria [5]. Tissue derived protein signals are products of a changing composition of viable, injured, and dead cells as well as infiltrating non-neural cells, that complicate the interpretation of proteomic studies [97,99]. An effort has been made in recent years to standardize and compare severities of commonly used TBI animal models across centers [123]. Defining common data elements for collection, analysis protocols, and reporting of fluid samples and histopathological defining features of injury models will help this field in interpreting proteomic and biomarker preclinical studies as well as clinical data collection and interpretation [124-126].

Proteomic TBI marker projects on biofluids using rodent injury models have been few due to naturally limited available fluid amounts [42,127], but biofluid neurotrauma marker candidates have been studied in pig blast injury models [128-132]. Human proteomic analyses have started from severe trauma patient’s CSF and plasma from individual patients [41,133]. Bioinformatics analysis tools are expected to facilitate systems level understanding of neurotrauma protein changes [134,135]. While bioinformatics tools are indispensable for classification, consensus-based data collection, and data mining, they will not make the bottleneck of biomarker candidate *selection* much easier.

Hanrieder *et al.* describes a workflow using matrix-assisted laser desorption ionization time-of-flight (MALDI-TOF) MS/MS in conjunction with off-line nano-LC sample fractionation [136]. In their study, ventricular CSF samples from 3 severe TBI patients displaying different symptoms were taken at various time points post-trauma and analyzed by nano-LC MALDI-TOF MS/MS to determine temporal protein expression changes. CSF samples were digested with trypsin and labeled with isobaric tags for relative and absolute quantitation using the iTRAQ method [137,138]. Labeled tryptic digests were then separated on a nano-flow LC system equipped with on-line fraction collection capable of depositing fractions directly onto MALDI sample plates for MALDI-TOF MS/MS-based identification and quantification. Several proteins were increased after injury. Additionally, relative quantification using iTRAQ labeling revealed temporal changes in protein expression for several inflammation-related proteins (e.g., serum amyloid, fibroinogen alpha chain, ceruloplasmin) as well as known neurotrauma-related proteins (GFAP, NSE).

Due to the confounding complexity of clinical TBI and clinic-resembling animal injury models, we propose a *targeted proteomic screen* using a well-characterized *in vitro* cell-based trauma model as a starting point for TBI marker candidate identification [139-144]. This will limit protein changes to those directly related to an acute mechanical trauma by applying an abrupt pressure pulse inflicting shear forces and deformation onto cortical brain cells in a reproducible fashion at various severities [142]. We are finding robust cellular release patterns that correlate with cell injury and cell death of rodent and human astrocytes matured and stretched in a prototype of this injury model [139] (Levine *et al*., submitted; manuscript in preparation). A suitable selection strategy needs to be applied to any trauma-release protein list to eliminate proteins found in healthy human plasma and to focus on brain-specific proteins [145,146].

### Verifying biochemical markers for TBI by proteomics and mass spectrometry

One analytical challenge that is unique to TBI for measuring candidate biomarkers lays in the unpredictably fluctuating protein concentrations among CSF samples from different TBI patients (low microgram/ml to several mg/ml range). This maybe due to as variables such as the patient’s varying blood–brain barrier integrity, hemorrhage, brain cell protein leakage, as well as waves of brain cell death. This is unlike healthy CSF or plasma with constant and physiologically controlled protein levels allowing for sample preparation with reproducible protein amounts [147]. These injury specific variables can be addressed only by relating all measurements to raw, unprocessed sample volume regardless of depletion and other processing steps including optimizing protein amounts for trypsin digestion or immunoassay applications. There are also injury-related but not-trauma specific secondary changes in protein composition in trauma CSF that could be caused by secondary infection due to hospitalization that could reduce protein amounts or bacterial proteins present in the samples. Such samples should be omitted from an initial biomarker validation study.

The accepted "gold standard" of single-protein measurements is the ELISA immunoassay, which takes advantage of the specificity and diversity of IgG antigen recognition. Yet, while ELISA is well touted for its high sensitivity (~1 pg/mL), it is not without limitations [148]. ELISA methods rely on antibodies for protein detection and assay development ideally uses two antibodies against different epitopes of the candidate TBI marker. Non-specific binding of immunoglobulins to abundant plasma proteins may contribute to a background problem, limiting the availability of suitable highly specific antibodies ideally from different host animals to cancel out non-specific binding. The availability of such antibody pairs often requires *de novo* generation, lengthening the assay development time. Thus, the lack of multiplex capacity may exclude using the ELISA platform as initial validation tool of candidate TBI markers in patient samples [149].

By not relying on antibody-antigen binding, quantitative mass spectrometry is well suited to meet the challenge of overcoming the verification bottleneck where immunoassays cannot be applied. MRM-MS is quickly becoming the preferred method of candidate biomarker verification because of the discriminating power of mass analyzers to accurately measure and quantify multiple specific proteins within a single sample set. Specific peptide fragments (via trypsin digestion) corresponding to the candidate proteins are selected to act as stoichiometric representatives (or surrogates) within a complex patient CSF or blood sample. The mass spectrometer (usually a triple quadrupole analyzer) is then set to scan for the precursor peptide ion, fragment the precursor in the collision cell, and then select for a specific precursor fragment (known as a transition). Because the mass spectrometer is not expending resources scanning through all the ions within a complex patient sample, the signal from less abundant peptides are no longer being masked by highly abundant ions, partially addressing the problems with high dynamic range limitations. Additionally, MRM provides a more cost-effective alternative for quantification compared to traditional ELISA methods by using stable isotope-labeled internal standards of the selective candidate peptides. Using the method of isotope dilution [150], isotopically labeled peptides are spiked into digested CSF or blood samples and the relative peak heights between the endogenous peptides and isotope-labeled peptides are used to quantify selected candidate biomarkers. This approach has been greatly aided by the increased availability of stable isotope-labeled standard (SIS) peptides manufactured and sold by life science companies [151]. MRM-MS has long been a method of choice for detecting marker metabolites for amino acids, organic acids, and fatty acid disorders in newborns [152]. The success of these quantitative methods in candidate biomarker discovery/verification has been well documented in a variety of samples such as synovial fluid [153], CSF [154], and plasma [155].

The MRM-MS platform is ideally suited to address the challenge of validating several marker candidates at once (multiplexing) and measuring their levels together with candidate TBI markers reported in the literature. This is in large part due to advances in in pre-analysis enrichment methods [156] as well as improvements in both sensitivity and speed of modern mass spectrometers that allow for detection and quantitation in the low-mid ng/mL concentration range. Hybrid orbitrap mass spectrometers such as the Q-Exactive have demonstrated the ability to detect up to 10 amol of heavy SIS peptides in the presence of 10 ng- 1 ug of yeast tryptic digest background with up to 10 ppm mass accuracy [157]. Coupled with the high resolving power of orbitrap detectors (up to 140 K for the Q-Exactive) and fast duty cycles to collect full MS/MS spectra, these instruments should be able to confidently identify surrogate peptides. When comparing the low cost of SIS peptide generation from commercial sources to the cost of antibody generation and capacity to multiplex more than ten within a single analytical sample, the mass spectrometry platform is a feasible choice for TBI candidate marker verification for the early preclinical validation stage. Following this initial verification, antibodies will be generated only for the most robustly detected TBI marker candidates for ELISA assay development for future clinical trials and diagnostic use.

## Conclusions

Combining a targeted screen, a focused selection strategy, and a stepwise approach from preclinical validation towards clinical translation offers a feasible pipeline for candidate TBI marker identification and preparation for its diagnostic use. Validation through a stepwise increasing sample cohort and moving from severe TBI CSF to matching plasma samples and then to mTBI plasma samples will provide verification where experimental analyses and patient samples are matched with appropriate positive controls along the way.

Moreover, the emergence of targeted MS-technologies brings promise to the development of an efficient biomarker discovery to verification pipeline for TBI. This pipeline could consist of the initial application of proteomics technologies in the form of comparative 2D-PAGE and shotgun LC-MS/MS to identify and discover candidate biomarkers from trauma and healthy subject samples. This is followed by the development of quantitative MRM-MS to assess the biological significance of these markers followed by clinical validation in a larger scale. With the possibility of multiplexing using proteomic methods such as MRM-MS, the time required for preclinical verification can be reduced as tens of marker candidate proteins can be monitored concurrently in clinical samples. This process will help narrow the pool of potential surrogates from which the most specific and easily measured candidates can be chosen for clinical validation and assay development.

## Competing interests

The authors declare that they have no competing interests.

## Authors’ contributions

SS, RROL, IBW, and JAL reviewed the relevant literature. All authors participated in the drafting of the manuscript, and all have read and approved the final manuscript.
